# Individual costs and community benefits: Collectivism and individuals’ compliance with public health interventions

**DOI:** 10.1371/journal.pone.0275388

**Published:** 2022-11-03

**Authors:** Suyi Leong, Kimin Eom, Keiko Ishii, Marion C. Aichberger, Karolina Fetz, Tim S. Müller, Heejung S. Kim, David K. Sherman

**Affiliations:** 1 Department of Psychological and Brain Sciences, University of California, Santa Barbara, Santa Barbara, CA, United States of America; 2 School of Social Sciences, Singapore Management University, Singapore, Singapore; 3 Department of Cognitive and Psychological Sciences, Graduate School of Informatics, Nagoya University, Nagoya, Japan; 4 Berlin Institute for Integration and Migration Research (BIM), Humboldt-Universität zu Berlin, Berlin, Germany; 5 Department of Psychiatry and Psychotherapy, Division of Social Psychiatry, Medical University Vienna, Vienna, Austria; University of Milan, ITALY

## Abstract

Differences in national responses to COVID-19 have been associated with the cultural value of collectivism. The present research builds on these findings by examining the relationship between collectivism at the individual level and adherence to public health recommendations to combat COVID-19 during the pre-vaccination stage of the pandemic, and examines different characteristics of collectivism (i.e., concern for community, trust in institutions, perceived social norms) as potential psychological mechanisms that could explain greater compliance. A study with a cross-section of American participants (*N* = 530) examined the relationship between collectivism and opting-in to digital contact tracing (DCT) and wearing face coverings in the general population. More collectivistic individuals were more likely to comply with public health interventions than less collectivistic individuals. While collectivism was positively associated with the three potential psychological mechanisms, only perceived social norms about the proportion of people performing the public health interventions explained the relationship between collectivism and compliance with both public health interventions. This research identifies specific pathways by which collectivism can lead to compliance with community-benefiting public health behaviors to combat contagious diseases and highlights the role of cultural orientation in shaping individuals’ decisions that involve a tension between individual cost and community benefit.

## Introduction

As the COVID-19 pandemic began to affect people around the world, and public health officials advocated policies to reduce the spread of the disease, people from different countries responded differently, and people *within* those countries responded with great variation as well. Cultural values have been useful to understand both this cross-national and within-nation variation–an issue with both theoretical and applied importance. For example, nation-level collectivism has been associated with greater adherence to public health interventions as several studies have found stronger compliance with public health interventions in more collectivistic countries as well as more collectivistic regions within the United States than less collectivistic countries and regions [1, 2]. Other cultural variables, such as cultural tightness-looseness, have also explained important COVID-19 outcomes [3]. Specifically, compared with “looser” countries (i.e., countries with greater tolerance for deviance), “tighter” countries (i.e., countries with greater emphasis on compliance and conformity) were associated with fewer COVID-19 positive cases and related death [3]. Although illuminating, the existing literature has focused on the association between cultural variables such as collectivism and compliance on the societal level, and thus, the understanding of whether and why collectivism is associated on an individual level with important outcomes such as adherence to public health recommendations remains an open question.

In the present paper, we sought to advance understanding of the psychological mechanisms underlying the association between collectivism and individuals’ likelihood to comply with two non-pharmaceutical public health interventions designed to reduce the spread of contagious diseases such as COVID-19—opting in to digital contact tracing and wearing face coverings. Considering collectivism as an individual-level value orientation, we tested three characteristics of collectivism as potential psychological explanations–concern for community, trust in government, and perceived social norms. We used a dataset collected in mid-2020 when pharmaceutical interventions, such as medication and vaccines, were not yet available to treat COVID-19.

### Collectivism and pathogen threat at the societal- and individual-levels

Cultural orientation shapes individuals’ views of themselves in relation to others, and influences individuals’ reactions and behaviors in social situations [4, 5]. Collectivism, both as a group- and individual-level factor, was characterized by the prioritization of communal goals over self-goals [5], and greater consideration of the consequences of one’s actions on their group members [6]. These collectivistic tendencies were particularly beneficial for the in-group, both psychologically and behaviorally, in the face of a common threat. For instance, historically, societies with greater levels of pathogen prevalence tend to be more collectivistic [7]. In the context of COVID-19, national- and U.S. state-level collectivism positively predicted wearing face coverings [1], and national-level, lower pathogen spread and mortalities, and greater adherence to prevention measures [8, 9].

Building on these findings with state- and country-level collectivism, we focused on collectivism and individualism as an individual-level value orientation within the United States. The influence of group- and individual-level cultural orientation on people’s behaviors was consistent yet independent [10, 11]. For example, a study that examined the role of individual- and U.S. state-level collectivism as a psychological buffer against the threat of a contagious disease found that both levels of collectivism showed consistent but independent influence [12]. Consistent with group-level findings, individuals endorsed stronger collectivistic values during, compared to before, the outbreak of COVID-19 pandemic [13]; and individuals with stronger collectivistic values were more likely to view social distancing as a form of disease prevention (e.g., agreeing more with items such as “the social distancing measure can help prevent me from getting the virus”), and were more likely to support social distancing practices (i.e., stay-at-home; 14).

In the present study, we investigated the role of the cultural dimension of collectivism, controlling for individualism. Individualistic and collectivistic value orientations have been shown to have distinct effects on behavioral outcomes [11], and prior work has shown that collectivistic-values orientation is a stronger predictor of responses to collective threats such as climate change [15] and contagious diseases [12, 16] than individualistic-values orientation. For example, in the face of Ebola, for people who were at high levels of perceived disease vulnerability, collectivism was associated with reduced xenophobic policy support, possibly because they perceived greater protection from their community against pathogen threat, whereas individualism was not associated with xenophobic policy support [12, 13]. In addition, collectivistic values orientation (but not individualistic values orientation) interacted with beliefs about climate change to predict climate action [15]. Not only is collectivistic values orientation a stronger predictor of response to collective threats, but people respond to collective threats by endorsing collectivistic values [7, 16]. In an examination of the difference in endorsement of collectivistic and individualistic values before and after the announcement of COVID-19 outbreak in South Korea, Na and colleagues found that individuals endorsed stronger collectivistic values, but there were no significant changes in the endorsement for individualistic values [13]. On days with higher daily confirmed cases, people tend to endorse more collectivistic values [13]. Together, these findings supported the distinct influence of individualistic and collectivistic-value orientations in the context of COVID-19, and led to the focus on collectivistic-value orientation would predict responses to collective threat.

In the present study, we posit that individual-level collectivistic values orientation functions similarly as has been shown at the group-level. Although several findings have demonstrated the positive association between collectivism and greater compliance with public health measures [1, 8, 13], there was a lack of understanding for *how* and *why* such positive associations exist. Thus, we proposed and tested three distinct psychological pathways–the potential roles of concern for community, trust in government, and perceived social norms–to explain the association between collectivism and greater compliance with public health measures.

#### Community concern

Given that some public health interventions incur a personal cost for a collective goal (e.g., inconvenience of wearing a mask), greater concern for in-group goals may explain why collectivists were likely to make community-serving decisions even if there was some personal cost. Numerous studies have demonstrated that people with greater collectivistic tendencies were more concerned about the impacts and consequences of their actions on their in-group, such as the community where they live, and were more likely to engage in behaviors that preserve group harmony [4, 5, 6]. In situations that involved sacrificing self-interest for a collective goal, more collectivistic individuals were more likely to sacrifice their self-gain and allocate more resources to benefit their in-group members [17]. A recent study on vaccination against influenza and COVID-19 revealed that prosocial concerns (e.g., worry of infecting others) predicted stronger vaccination intentions when social density was low (e.g., rural areas in the U.S., where people were likely more collectivistic), presumably because individuals viewed that their behaviors had greater impacts on others [18]. Thus, given the greater emphasis on in-group goals and priorities, we posit that people higher on collectivism may comply with non-pharmaceutical interventions (NPIs) to prevent the spread of COVID-19 to a greater extent due to greater concern for community wellbeing.

#### Trust in government

Combatting infectious diseases requires a collective effort and public compliance with measures spearheaded by an authority, oftentimes the government. Public compliance with COVID-19 policies was greater in many Asian countries, possibly because of collectivists’ stronger general tendency to follow authorities’ recommendations [19]. As evidenced in pandemics current and past, individuals with greater trust in government were more likely to follow health guidelines and engage in preventive behaviors [20, 21]. Americans who scored higher in authoritarianism, a personality trait associated with dogmatism and support of strong governmental policies, were more in favor of mandatory digital contact tracing in response to COVID-19, compared to those low in authoritarianism [22, 23]. More generally, compared to more individualistic people, collectivistic people tend to make decisions that reflect deference to authority and they experience greater guilt if they behave in a way that violates the expectations of authorities [24]. Further supporting this pathway, a cross-cultural study that examined the role of trust in government and the relationship between cultural orientation and compliance to health measures revealed that collectivism was associated with stronger trust in government [25]. Taken together, we posit that collectivists’ trust in authority as benevolent and efficacious may be one reason why they more closely follow public health recommendations.

#### Perceived social norms

Social norms were important and powerful factors that shape people’s behaviors, especially in more collectivistic cultural contexts [26]. There were at least two possible pathways through which people in different cultures respond to social norms. The first pathway centered on cultural differences in the perception of norms. Collectivists made fewer distinctions between self and others, and viewed personal and group identities as interchangeable [27]. Indeed, prior studies have demonstrated that while both collectivistic and individualistic individuals overestimate the percentage of people who agree with their opinions (i.e., exhibit false consensus effect; 28) the effect was stronger among collectivists relative to individualists [29, 30]. Thus, collectivists may project their own behaviors to the group and use group behaviors to inform their own.

The second path is through conformity. Conformity to social norms has been shown to drive a wide range of behaviors that could be in the public interest such as fostering adaptive health behaviors [31]. In terms of COVID-19 preventive behaviors, for example, individuals were more likely to wear a mask when a great proportion of people in their proximity are also wearing masks [32]. Although social norms were powerful in shaping people’s behaviors generally, in more collectivistic cultural contexts, conformity and behaving in ways consistent with the group were more prevalent and valued, compared to more individualistic cultural contexts [33, 34]. Thus, when clear norms were present, people from collectivistic cultural contexts were more likely to conform, compared to people from individualistic cultural contexts [26].

Both paths could potentially explain how collectivistic people engage in coordinated collective actions, compared to less collectivistic people, in a real-life situation where social norms rapidly and dynamically form such as the occurred during the beginning of COVID-19 pandemic. The first path assumed that perceived social norms can serve as a mediator, such that more collectivistic individuals will report greater perceived proportion of people who complied with public health behaviors, and in turn, greater perceived social norms will predict greater compliance with the targeted health behavior. The second path, through conformity, assumed that collectivism may strengthen the relationship between perceived social norms and compliance, treating collectivism as a moderator [10, 15]. Therefore, in the present study, we conducted both mediation and moderation analyses to parse the influence of perceived social norms on compliance as a function of collectivism.

#### Cultural tightness-looseness

In this study, we also consider the potential role of cultural tightness-looseness. Tightness-looseness was operationalized by the strength of social norms both within and between cultural contexts [35]. At the group-level, countries with tighter cultures (e.g., China, Singapore) enforced stronger social norms and greater punishment for deviance, while countries with looser cultures (e.g., United States, Brazil) had weaker norms and more tolerance for deviance [35]. Within the United States, state levels of tightness are positively correlated with state level collectivism index [36], *r* (50) = .23, *r* (49) = .37 omitting Hawaii, although they are clearly differentiable constructs [37]. On the individual-level, people who perceived that their states had tighter norms were more likely to engage in COVID-19 prevention behaviors [38]. Moreover, there were some parallel findings with those reported earlier showing the relationship between collectivism and COVID-19 outcomes [1]. Tighter countries had lower COVID-19 positive rates and mortality, while looser countries had higher positive rates and mortality [3]. Given these findings, and the importance of social norms to both constructs, although our primary theoretical focus was on collectivism, we also assessed tightness-looseness and controlled for it in subsequent analyses.

### Public health behaviors to combat COVID-19

Addressing threats to public health from contagious diseases requires the adoption of behaviors, whether pharmaceutical (e.g., vaccine) or non-pharmaceutical interventions (e.g., mask wearing, social distancing, and contract tracing) by individuals within the society. For these approaches to be successful, a society will need a great majority of its citizens to be willing to comply.

The present study focused on two key public health related behaviors, digital contact tracing (DCT) and wearing a face covering. DCT effectively aided traditional contact tracing efforts by identifying potential exposure to a virus based on the location and duration of interactions between two (or more) people through cellular technology; wearing face coverings created a barrier to prevent respiratory droplets from reaching others [39]. Moreover, we proposed that these behaviors provided a particularly relevant context to examine the relationship between collectivism and compliance. First, although the benefits of these behaviors exist at both personal and group-levels, opting-in to DCT and wearing face coverings imposed some individual costs to achieve a collective goal. Individuals may view DCT as a violation of their privacy, a new form of government surveillance [40, 41], or a potential source of discrimination and stigmatization (e.g., fear of being judged, 40). Wearing face coverings can be uncomfortable and inconvenient, and was associated in some contexts with the stigma of being sick and weak [42–45], and was viewed as a violation of personal liberty [46]. Second, both measures required a sufficient proportion of the population to comply to be optimally effective. For example, at least 60% of the population has to opt in for digital contact tracing to be effective [47]. Similarly, immediate or near universal face covering usage (> 80%) could have decreased death and positive rates substantially from COVID-19 [43]. Although evidence demonstrated the positive association between collectivism and compliance with health measures at the country level [1], understanding how and why individuals comply at the individual level was important in order to carry out successful community-level public health interventions.

Yet, there were also several key features that differentiate opting-in to DCT from wearing face coverings in public that facilitate tests of the generalizability of the model. First, these two public health behaviors vary in the extent to which performance was signaled to others publicly. In most contexts, opting-in to DCT was a private decision that only involved downloading an app to one’s phone without the need to inform anyone, whereas wearing face coverings was an inherently publicly visible behavior. Second, while health authorities have strongly recommended and even mandated the use of face coverings in public, opting-in to DCT has been voluntary, and not widely implemented. Third, participants’ intentions to opt in to DCT was only hypothetical at the time of data collection due to no official implementation of DCT in the U.S., but wearing a face-covering in public was actively practiced. Thus, inclusion of both outcome variables allowed us to test the generalizability of the theoretical model. With these target behaviors, we examined whether and why collectivism as an individual value orientation played a significant role in influencing people’s decisions to comply with these two public health behaviors.

There were two primary objectives in this study. First, we seek to examine the role of individual-level collectivism in people’s decisions to comply with NPIs intended to prevent the spread of COVID-19. We hypothesized that more collectivistic people would be more likely to comply with NPIs than less collectivistic people. Second, we explored potential psychological mechanisms that explain greater compliance with NPIs among those who are more collectivistic. We tested three potential mediators–concern for community, trust in government, and perceived social norms–to explain the relationship between collectivism and compliance. The present study included measures of individualism-collectivism, three potential mediators, and self-report compliance with two public health behaviors, opting in to DCT and wearing face coverings. We included several demographic covariates as well as tightness-looseness to examine the robustness of the findings. All analyses were conducted in SPSS 28, and PROCESS Macro [48]. Data, code, and materials for the study including additional scales for other research purposes not used in the present analyses are available on OSF (https://osf.io/ganem/).

## Method

A total of 530 (In Sherman et al. (2022), there was a correlation of *r* = .13 between collectivism and compliance with environmental behaviors. Power analyses conducted in G*Power revealed that a correlation of this magnitude could be obtained with .80 power and an *α* = .05 with *N* = 462.) participants were recruited through Amazon Mechanical Turk. Data was collected between July 1 and July 17, 2020. 37.4% of the participants identified as female, 57.5% as male, 0.6% as non-binary/other, and 4.5% unspecified. The mean age of participants was 37.21(*SD* = 11.28). 68.1% of the participants identified themselves as White, 13.4% as Black, 6.4% as Hispanic/Latino, 5.5% as Asian, 0.4% as American Indian, 0.2% as Native Hawaiian or Pacific Islander, and 1.6% as multi-racial/others. The remaining participants did not identify their racial/ethnic identities. Refer to Table 1 for full demographic information.

**Table 1:** Participants demographic characteristics.

Characteristics		M(SD)	n	%
Age		37.21(11.28)		
Years of Education		14.58(4.30)		
Income (Median)		$40,000 - $49,999		
Political Ideology		3.86(1.88)		
Gender				
	Male		305	57.5
	Female		198	37.4
	Other		3	.6
	Missing		24	4.5
Ethnicity				
	American Indian/Alaska Native		2	.4
	Asian/Asian American		29	5.5
	Black/African American		71	13.4
	Hispanic/Latino American		34	6.4
	Native Hawaiian/Pacific Islander		1	.2
	White/European American		361	68.1
	Other/Unspecified		8	1.5
	Missing		24	4.5

### Procedures

The survey was conducted online in July 2020, when COVID-19 positive rates were increasing exponentially in the United States. The survey was conducted in English language. At the start of the survey, participants provided their written consent to take part in the study through selecting the “Yes, I agree to participate” option after reading a consent form that contained study information. After indicating their willingness to take part in the study, participants responded to measures of cultural orientation (e.g., individualism-collectivism, tightness-looseness). Then, they read a short passage about what digital contact tracing is, and how it works, and answered some questions about their attitudes and intention to opt in to DCT. In the second part of the study, participants reported their attitudes towards face covering, and indicated whether they wear a face covering when it is required, and when it is not required. Participants responded to the mediator variables after responding to the outcome variables (i.e., decision to opt-in to DCT and wear a face covering). Lastly, participants reported their demographic information. All participants were debriefed in writing at the end of the survey. The survey took approximately 12 minutes to complete, and participants were compensated $1.50. This study was reviewed and approved by UCSB Office of Research Application for the use of Human Subjects.

### Materials and measures

#### DCT information

Participants were provided with the following information about DCT:

“What is contact tracing? Contact tracing for COVID-19 is the process of identifying, assessing, and managing people who have been exposed to the disease to prevent onward transmission. Digital contact tracing tools aid traditional contact tracing efforts by using data from people’s mobile phones. How does digital contact tracing work? To use digital contact tracing tools, people download an app on their mobile phones. When someone tests positive for COVID-19 and shares this information via the app, the app automatically and anonymously notifies other people who had contact with the person. These individuals with potential exposure are advised to be tested and/or quarantined.”

#### Predictor variable

*Individualism-collectivism*. Participants completed a 14-item validated individualistic and collectivistic value orientation measure [11, 12]. This scale has been used in similar samples as the present paper in Kim and colleagues [12], and Sherman and colleagues [15]. Individualism items included “it is important for me to develop my own personal style,” while collectivism items included “it is important for me to think myself as a member of my religious, national, or ethnic group.” All items were assessed on 7-point scales ranging from 1 (strongly disagree) to 7 (strongly agree). Individualism and collectivism items were averaged and each formed a composite score, with higher values indicating higher endorsement of each cultural value orientation (Individualism: *M* = 4.94, *SD* = 1.19, *ɑ =* .86; Collectivism: *M* = 5.65, *SD* = .81, *ɑ* = .76). See Table 2 for descriptive statistics and alpha levels for all key measures.

**Table 2:** Descriptive statistics for key variables.

	M	SD	*ɑ*
Collectivism	4.94	1.19	.86
Individualism	5.65	.81	.76
Tightness-Looseness	5.35	.81	.67
Trust in Government	2.82	1.09	.75
Concern for Community (DCT)	61.53	28.44	-
Perceived Social Norms (DCT)	58.99	24.84	-
Concern for Community (FC)	61.96	30.32	-
Perceived Social Norms (FC Required)	73.91	19.40	-
Perceived Social Norms (FC Not Required)	59.68	26.23	-

*Tightness-looseness*. Participants responded to six items that measured culture tightness-looseness [35]. The tightness-looseness measure has been developed by Gelfand and colleagues [35] and has been used across many settings, and shown to be reliable in the study among 33 nations sampled (see also 49). An example item was “There are many social norms that people are supposed to abide by in this country.” All items were assessed on a 7-point scale ranging from 1 (strongly disagree) to 7 (strongly agree), with higher values indicating greater tightness, *M* = 5.35, *SD =* .81, *ɑ =* .67.

#### Outcome variables

Dichotomous variables were created to operationalize the choices that people make in terms of compliance with COVID-19 public health behaviors. We developed these dichotomous outcome variables as they were highly context specific (i.e., these behaviors were more prominent because of the pandemic), and did not have previous scales to adapt from. Similar measures, however, have been used in Lu and colleagues [1] in the context of COVID-19 preventative behaviors.

*DCT Decision*. Participants responded to the question “if your health authority administers digital contact tracing, would you opt in (sign up for the app) or opt out (not sign up for the app)?” to indicate their intention to opt in to DCT (0 = I would opt out; 1 = I would opt in).

*Face Coverings (FC)*. Participants responded to two dichotomous questions that assess two different situations where people may wear masks, where it was required and when it was not required. Specifically, they responded (0 = No, 1 = Yes) to the queries: “do you use face covering where it is required?”, and “do you generally use face covering even when it is not required (in social places where you interact with other people)?”.

#### Mediator variables

*Concern for community*. Although participants may comply with these NPIs to protect both themselves *and* their community health, we created single-item measures to examine participants’ primary motivation in their decision-making process for each behavior. Participants responded to the question “in considering digital contact tracing, which factor is more important to you?” Participants rated on a sliding scale from 0 (*protecting myself from COVID-19*) to 100 (*protecting my community from COVID-19*), with higher values indicating the tendency to prioritize the community’s health over oneself, *M* = 61.53, *SD* = 28.44. The same question was posed for wearing face coverings. Participants responded to the question “in considering wearing a face covering, which factor is more important to you?” Participants rated on a sliding scale from 0 (*protecting myself from COVID-19*) to 100 (*protecting my community from COVID-*19), *M =* 61.96, *SD* = 30.32. These measures provided continuous assessments of participants’ primary motivations for action.

*Trust in government*. Participants completed four items that were adapted from the trust in government survey by Pew Research Center [50], which is an extensive battery of questions that Pew asks on an annual basis to gauge Americans trust in different aspects of the government. We chose items that we thought would capture the varying perceptions Americans had of the government as it was making decisions during the COVID-19 pandemic. The four items were: “I generally think the government is run for the benefit of all the people in this country.”; “Most of the time I think I can trust the Government in Washington DC to do what is right”; “I generally think the government is run for the benefit of all the people in this country”; “I think the people in the government waste a lot of money we pay in taxes”; and “In my opinion, quite a few of the people running the government are crooked”. All items were assessed on 6-point scales ranging from 1 (*strongly disagree*) to 6 (*strongly agree*). Similar measures have also been used in Travaglino & Moon [25], also in the context of the governmental response to COVID-19. Items were averaged and formed a composite score, with higher values indicating greater trust in government, *M =* 2.82, *SD* = 1.09, *ɑ =* .75.

*Perceived social norms*. We assessed participants’ perceptions of the proportion of people in their communities who engage in the different public health behaviors using a measured developed by Eom and colleagues [10]. Participants indicated on a 0 to 100% sliding scale the proportion of people in their community who they think would opt in to DCT, and the proportion of people in their community who they think would wear face coverings when is required, and when it is not required; with higher values indicating a larger perceived proportion of people in their community who comply with these health measures, DCT: *M =* 58.99, *SD* = 24.84; FC (Required): *M =* 73.91, *SD* = 19.40; FC (Not Required): *M =* 59.68, *SD* = 26.23.

#### Covariates

*Demographics*. We controlled for participants’ gender, age, income, and political ideology. Given the political sentiment associated with COVID-19 related attitudes and behaviors [51–53], we controlled for participants’ political ideology to assess the robustness of collectivism in predicting compliance. Participants responded to the question “when it comes to politics, do you consider yourself to be liberal, moderate, or conservative?” as a measure of their political ideology. The question was assessed on a 7-point scale ranging from 1 (*very liberal*) to 7 (*very conservative*).

### Results

#### Compliance with NPIs

Overall, 65.1% of participants indicated that if offered by their health authorities, they would opt in to digital contact tracing, whereas 34.9% would opt out. In terms of their current face-covering behavior, almost all participants (96.0%) reported that they use face covering when it is required. 77.2% participants reported that they still use a face covering, even if it was not required in places where social interactions took place. For analyses, then, we focused on the decision to wear face covering when it is not required. Refer to Tables 3 and 4 for zero-order correlations between key variables.

**Table 3:** Zero-order correlations between key variables related to digital contact tracing (DCT).

	COL	IND	TL	Concern for Community	Trust in Gov.	Perceived Social Norms	DCT Decision
Collectivism	-						
Individualism	.39**	-					
Tightness-Looseness	.47**	.45**	-				
Concern for Community	.35**	.09	.20**	-			
Trust in Gov.	.52**	.04	.20**	.32**	-		
Perceived Social Norms	.45**	.20**	.30**	.46**	.47**	-	
DCT Decision	.25**	.03	.12**	.27**	.31**	.43**	-

*p < .05

**p < .01

***p < .001

**Table 4:** Zero-order correlations between key variables related to face covering (FC).

	COL	IND	TL	Concern for Community	Trust in Gov.	Perceived Social Norms	Face Covering
Collectivism	-						
Individualism	.39**	-					
Tightness-Looseness	.47**	.45**	-				
Concern for Community	.29**	.01	.20**	-			
Trust in Gov.	.52**	.04	.20**	.31**	-		
Perceived Social Norms	.52**	.13**	.28**	.38**	.41**	-	
Face Covering	.10*	.02	.08	.12**	.06	.22**	-

*p < .05

**p < .01

***p < .001

We conducted a binary logistics regression and controlled for individualism, tightness-looseness, gender, age, majority group status, political orientation, annual income, and years of education (As Whites/European Americans have been shown to be less collectivistic in their value orientation than other groups (Oyserman et al., 2022), we also conducted analyses that controlled for race/ethnicity (coded as White/European Americans vs. racial/ethnic minority Americans). This variable was not a significant predictor of the key DVs and the direction and magnitude of findings remain unchanged when it is included.). Collectivism significantly predicted DCT opt in rates, *β* = .63, *SE* = .13, *p* < .001, and participants’ likelihood of wearing face covering when not required, *β* = .35, *SE* = .14, *p* = .02. More collectivistic participants were more likely to opt in to DCT and to wear a face covering when it was not required. Tightness-looseness, on the other hand, was not associated with greater likelihood of opting-in to DCT or wearing a face covering when it was not required (Table 5).

**Table 5:** Collectivism predicts compliance to health measures.

	Variable	*β*	*SE* of *β*	*Wald*	*p*	Exp (*β)*	95% CI	
							LL	UL
Digital Contact Tracing	Constant	1.30	.54	5.87	.02	3.68		
	Collectivism	.63	.13	25.17	< .001	1.88	1.47	2.40
	Individualism	-.22	.12	3.71	.05	.80	.64	1.00
	Tightness-Looseness	.06	.12	.28	.60	1.06	.84	1.34
	Gender (Male)	-.10	.21	.22	.64	.91	.61	1.36
	Gender (Female)	-.95	1.27	.55	.46	.39	.03	4.71
	Age	-.004	.01	.19	.67	.99	.98	1.01
	Political Ideology	-.14	.06	5.63	.02	.87	.78	.98
	Income	.09	.04	5.71	.02	1.10	1.02	1.18
	Years of Education	-.03	.02	1.55	.21	.97	.93	1.02
Face Covering	Constant	2.33	.61	14.13	< .001	10.25		
	Collectivism	.35	.14	6.53	.01	1.42	1.09	1.86
	Individualism	-.15	.13	1.14	.23	.86	.67	1.10
	Tightness-Looseness	.14	.13	1.15	.28	1.15	.89	1.49
	Gender (Male)	-.15	.23	.40	.53	.86	.55	1.36
	Gender (Female)	-1.99	1.26	2.48	.12	.14	.01	1.63
	Age	-.002	.01	.03	.86	.99	.98	1.02
	Political Ideology	-.33	.07	22.41	< .001	.73	.63	.83
	Income	.16	.05	11.76	< .001	1.17	1.07	1.28
	Years of Education	-.03	.02	1.43	.23	.97	.93	1.02

* *p* < .05

** *p<* .01

***p < .001

† R^2 DCT = .12, R^2 FC = .13

We conducted mediation analyses controlling for the same covariates for each of the two health measures to test whether concern for community health in relation to each health behavior, trust in government and perceived social norms explain the relationship between collectivism and compliance. Refer to Tables 6 and 7 for full regression coefficients.

**Table 6:** Regression coefficients for mediation models (DCT).

					95% CI of β	
	*β*	*SE of β*	*z*	*p*	*LL*	*UL*
Direct Effects						
COL → DCT	.23	.15	1.57	.12	-.06	.51
Separate Effect Paths						
COL → concern for comm.	.32	.05	6.22	< .001	.22	.42
COL → trust in gov	.51	.05	11.13	< .001	.42	.60
COL → perceived social norms	.34	.05	7.16	< .001	.25	.44
Concern for comm. → DCT	.15	.12	1.24	.22	-.09	.37
Gov. → DCT	.33	.13	2.43	.01	.06	.59
Norm → DCT	.82	.14	5.99	< .001	.55	1.09
Bootstrapped Indirect Effects	*β*	*BootSE*	*Boot LLCI*	*BootULCI*		
Total Indirect Effect of COL	.50	.10	.33	.74		
Concern for Comm	.05	.04	-.03	.14		
Trust in Gov	.17	.07	.04	.33		
Perceived Social Norms	.28	.08	.16	.47		

* *p* < .05; ** *p <* .01; ***p < .001

**Table 7:** Regression coefficients for mediation models (FC).

Digital Contact Tracing					95% CI of β	
	*β*	*SE of β*	*z*	*p*	*LL*	*UL*
Direct Effects						
COL → FC	.18	.16	1.13	.26	-.13	.48
Separate Effect Paths						
COL → concern for comm.	.31	.05	5.71	< .001	.20	.40
COL → trust in gov	.51	.05	11.12	< .001	.42	.60
COL → perceived social norm	.36	.05	7.41	< .001	.27	.46
Concern for comm. → FC	.06	.12	.52	.60	-.18	.31
Gov. → FC	-.07	.14	-.52	.60	-.34	.20
Norm → FC	.60	.13	4.53	< .001	.34	.86
Bootstrapped Indirect Effects	*β*	*BootSE*	*Boot LLCI*	*BootULCI*		
Total Indirect Effect of COL	.20	.09	.03	.39		
Concern for Comm	.02	.04	-.06	.10		
Trust in Gov	-.04	.07	-.17	.10		
Perceived Social Norms	.22	.06	.12	.36		

* *p* < .05; ** *p <* .01; ***p < .001

#### DCT opt in

**Fig 1:**
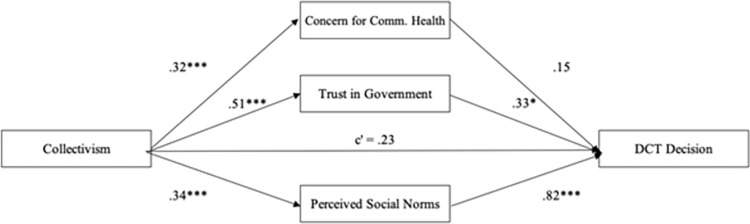
Mediation model for DCT decision. The relationship between collectivism and decision to opt-in to DCT as mediated by concern for community health, perceived social norms, and trust in government. Numbers are standardized regression coefficients.

In relation to the intention to opt in to DCT, collectivism predicted greater concern for community health, *β* = .32, *SE* = .05, *p* < .001, greater trust in government. *β* = .51, *SE* = .05, *p* < .001, and greater perceived social norms, *β* = .34, *SE* = .05, *p* < .001. Those who were more collectivistic had greater concern for their community’s health, felt greater trust in their government, and saw a greater proportion of other people as likely to opt in to DCT (Fig 1). In turn, greater trust in government, *β* = .33, *SE* = .13, *p* = .01, and greater perceived social norms, *β* = .82, *SE* = .14, *p* < .001, but not greater concern for community health, *β* = .15, *SE* = .12, *p* = .22, predicted greater likelihood of opting-in to DCT. Consequently, trust in government and perceived social norms each mediated the effect of collectivism on opting-in, as indicated by significant indirect effects (trust in government.: *β* = .17, *BootSE* = .07, BootCI[.04, .33]; perceived social norms: *β* = .28, *BootSE* = .08, BootCI[.16, .47]). By contrast, greater concern for community health was not a significant mediator (*β* = .05, *SE* = .04, BootCI[-.03, .14]). After controlling for all mediators, the association between collectivism and DCT opt in was non-significant, *β* = .23, *SE* = .15, *p* = .12.

#### Face covering

**Fig 2:**
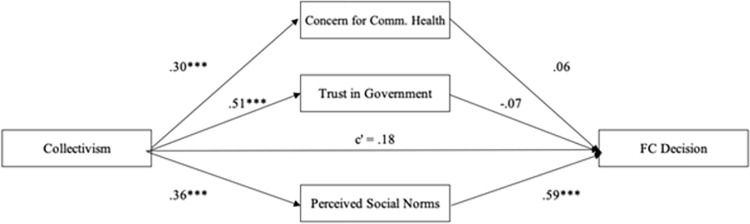
Mediation model for FC decision. The relationship between collectivism and compliance with wearing a face covering when not required as mediated by concern for community health, perceived social norms, and trust in government. Numbers are standardized regression coefficients.

The mediational pattern for wearing face coverings in public was somewhat different than for opting in to DCT (Fig 2). Collectivism was positively associated with all three mediators, as it was predicted greater concern for community health, *β* = .31, *SE* = .05, *p* < .001, greater trust in government, *β* = .51, *SE* = .05, *p* < .001, and greater perceived social norms, *β* = .36, *SE* = .05, *p* < .001. However, only greater perceived social norms predicted greater likelihood of wearing face coverings in public, *β* = .60, *SE* = .13, *p* < .001. Neither concern for community health, *β* = .06, *SE* = .12, *p* = .52, nor trust in government, *β* = -.07, *SE* = .14, *p* = .60, explained the relationship between collectivism and wearing face covering. Consequently, only perceived social norms mediated the effect of collectivism on wearing a face covering (*β* = .22, *BootSE =* .06, BootCI[.12, .36]). Neither concern for community health (*β* = .02, *BootSE =* .04, BootCI[-.06, .10]), nor trust in government (*β* = -.04, *BootSE =* .07, BootCI[-.17, .10]) were significant mediators. After controlling for all mediators, the association between collectivism and intention to opt in to DCT was non-significant, *β* = .18, *SE* = .16, *p* = .31.

#### Additional analysis: Does collectivism moderate the relationship between perceived social norms and compliance with health measures?

To examine whether collectivism affects the strength and direction of the relationship between perceived social norms and the decisions to comply with each health measure, we performed a moderation analysis using Hayes PROCESS Model 1 [48], controlling for individualism, tightness-looseness and other relevant demographic variables. Overall, the interaction between collectivism and perceived social norms did not predict greater likelihood of opting in to DCT, *β* = -.026, *SE* = .12, *p* = .83, or wearing a face covering when it is not required, *β* = -.17, *SE* = .12, *p* = .16. The relationship between perceived social norms and compliance with health measures was not moderated by individuals’ collectivism.

### Discussion

Collectivism, as assessed at the individual level as a cultural value orientation, significantly predicted adherence to public health interventions during the height of the COVID-19 pandemic, a time before vaccination where non-pharmaceutical interventions were the best methods of protecting public health. The relationship was strong and robust to controlling variables such as individualism, tightness-looseness, political ideology, and demographic factors such as race and SES. These findings advance the understanding of the relationship between cultural value orientations and behaviors that have some individual cost but the potential for community benefit. Using the pandemic as a ground for us to examine hypotheses, we found that even within a highly individualistic country like the United States, people can still exhibit collectivistic values, and among those who exhibit greater collectivistic values, they are more likely to comply with public health recommendations. We probed this research question further by examining *how* and *in what ways* collectivistic values shape people to comply. Although we recognize that culture-level collectivism is not the same as collectivism as an individual difference factor, the present research can advance understanding of the process of how collectivism shapes behaviors and presents ideas for future research.

In terms of understanding the psychological processes underlying this relationship, we examined three possibilities, and found that greater perceived social norms appeared to explain the relationship between collectivism and compliance to both public health recommendations. Those who are high in collectivism perceived that a greater proportion of people in their communities would engage in these behaviors, and this increased perception that the behaviors were normative predicted their own behavior. A second potential factor examined—greater concern for community health—was not a significant mediator for either behavior. Finally, there was variability across behaviors in the third mediator, as greater trust in government only explained the relationship between collectivism and opting-in to DCT but did not explain the relationship between collectivism and face covering behavior.

There are two reasons that may explain the inconsistent mediating patterns between opting-in to digital contact tracing and wearing face coverings in public. First, opting-in to DCT is a much more private decision, while wearing a face covering is directly observable. That only perceived consensus with others predicted greater likelihood of wearing face coverings suggested that people’s decisions to wear a face covering may be largely shaped by the behaviors of people around them (see also 32), but not necessarily their own concern for community health. Furthermore, although there was a positive association between collectivism and trust in government, our results suggest that trust in government has greater importance for people’s decision to opt in to DCT, but not wearing a face covering in public. One possibility is that government directly implements programs such as digital contact tracing, whereas face covering, in particular in places where it is not required, is an individual choice.

#### Theoretical implications

The present study contributes to the literature in several ways. First, the association between collectivism and compliance with both public health behaviors–above and beyond other cultural values, tightness-looseness, political ideology, and demographic factors–builds on the studies that examined compliance at the state and national levels [1]. The robustness of collectivism as a predictor at both the collective- and individual-level highlights the role of culture in the making of health decisions that may impose some personal cost for a collective good.

Second, the present study examined how different aspects of collectivism shaped compliance. Across both behaviors, greater concern for community health did not explain the relationship between collectivism and compliance with public health behaviors. Perhaps one reason why community concern was not associated with compliance was due to the way we measured this item. Opting in to contact tracing and wearing face coverings provide protection for both personal health as well as community health. While the purpose of putting personal and community health at two ends of a continuum was to enable a test of which was a stronger factor that shaped people’s decisions, we acknowledge that this way of measuring this mediator overlooked the possibility that people comply with public health interventions to protect *both* their personal *and* community health. One way to refine this measure is to separate personal and community health into two distinct items. Nevertheless, the results suggest that more collectivistic individuals do not comply with these interventions solely or primarily because they are *more* concerned about their communities.

In contrast, perceived social norms appeared to be a strong mediator for compliance with both public health interventions in the present study. Without knowing the actual statistics of local community members who complied with NPIs, our study provided consistent evidence with existing research that demonstrated that collectivists tend to overestimate consensus with their in-group members [54]. Specifically, those high on collectivism perceived that a greater proportion of people in their local community complied with NPIs than those low on collectivism, and in turn, they were more likely to comply themselves. We also tested a viable alternative model whereby collectivism made people more attuned to the social norms, whether they are high or low by testing collectivism as a moderator of the relationship between social norms and compliance [10, 15]. There was no significant interaction between collectivism and perceived social norms on compliance behaviors, suggesting that perhaps collectivistic individuals comply not because of the contextual forces around them (i.e., strong norms in the environment), but rather, because of their own perception of what other people might do (i.e., intersubjective norms; see 55 for more discussion). Future studies should explore the role of intersubjective norm model in adherence to public health recommendations, where individuals rely on what they think others are doing as a means of guiding their own behaviors [55, 56].

Furthermore, the present findings suggest that greater trust in government may be an additional underlying mechanism that shapes people’s compliance. We found a positive association between collectivists’ trust and deference to government or authority (consistent with 25). However, the mixed patterns between opting-in to DCT and wearing a face covering suggest that the extent to which people’s trust in government translates to actual compliance is also conditional on the behavior under consideration. Given that DCT is a tool that needs to be implemented, in part, by the government, establishing trust in government is particularly essential to encourage greater compliance [57]. By contrast, for wearing a face covering, a behavior that is more visible among peers and communities, trust in government may not be a salient factor in individuals’ decisions, in particular, when individuals received mixed recommendations from the government during the onset of the pandemic [58].

Finally, in the face of a common threat such as disease pathogens, prior research has revealed the psychological benefits of collectivism such as providing greater protection efficacy [12]. Findings from the present study suggest that collectivists may feel more efficacious against threat by placing greater trust in authorities (see also 22), and perceiving greater social norms of compliance among their in-group members. A greater orientation towards others that fosters group coordination may also explain why individuals become more collectivistic in the face of a common threat (e.g., pathogen, 7)

### Limitations

There are several limitations in this study. First, the nature of this study is correlational, and we are unable to claim a causal role for either of collectivism or the mediators. We sought in our analyses to control for different demographic and political variables, as well as individualism and tightness-looseness to isolate the role of collectivism. Nevertheless, the limitations of correlational analyses are applicable here. Moreover, whereas the present study explores different aspects of collectivism, we also recognize that individuals’ engagement in public health behaviors are determined by other factors as well, both individual (e.g., perceived vulnerability to COVID-19) and institutional (e.g., sanctions for violation of policies).

Second, the decision to opt in to DCT was hypothetical. That is, given that there was no official implementation of DCT in the U.S., we measured participants’ intention to opt in. Although we included wearing face coverings as a (self-report) measure of actual ongoing behaviors, it is important to examine whether and how these intentions turn into actual opt in behaviors. We acknowledge that a limitation for self-report data is the inability to evaluate people’s actual behaviors. That is, although a relatively high proportion of participants (77%) reported wearing a face covering when it was not required, we were not able to objectively measure their actual behaviors, and self-reports are necessarily strongly related to actual behaviors (see 59 for recent discussion). Despite these limitations, findings from our studies were consistent with those that examined group-level collectivism and compliance using regional, aggregated mask usage as a dependent variable [1], demonstrating the robustness of collectivism as a predictor for compliance across different levels of analyses.

Third, the study was a cross-sectional study conducted in the United States, where the handling of the pandemic may be vastly different from other countries (although there does seem to be common psychological responses to public health policies; see 60). Even within the United States, each state has their own health regulations, and variability such as mask mandates (or lack thereof), and the spread of COVID-19 are factors that may have influenced how participants reacted to these health measures [1]. The cross-sectional nature of the study also limited our ability to understand the changes in compliance as the pandemic progressed. Despite all these differences, we found a robust positive association between collectivism and compliance with these public health measures.

### Conclusions

Even within a highly individualistic cultural context such as the United States, the present research demonstrates how individual-level collectivism can be powerful in encouraging compliance with public health interventions. The benefit of identifying psychological mediators is that it points to additional levers, such as increasing the perception of public norms and fostering trust in government that can be pulled to facilitate compliance in situations that involve tension between personal costs and collective benefits [21, 61]. Furthermore, public health campaigns could leverage people’s tendencies to exhibit greater collectivism [7, 13], as well as the buffering effect of collectivism in the face of a common threat [12] by emphasizing group cohesion and reminding people of their close others. As the Director-General of the World Health Organization put it, “COVID-19 has…shaken the foundations of our world; …but it has also reminded us that for all our differences, we are one human race. And we are stronger together [62].” The relevance of this message goes beyond the current pandemic and the polarized times we are living through. Such invocations of togetherness may be an essential ingredient for coordinated human efforts to combat many threats and challenges that humans face in the future.
